# Insulin-like growth factor 2 mRNA-binding proteins (IGF2BPs): post-transcriptional drivers of cancer progression?

**DOI:** 10.1007/s00018-012-1186-z

**Published:** 2012-10-16

**Authors:** Jessica L. Bell, Kristin Wächter, Britta Mühleck, Nikolaos Pazaitis, Marcel Köhn, Marcell Lederer, Stefan Hüttelmaier

**Affiliations:** Section for Molecular Cell Biology, Institute of Molecular Medicine, Martin-Luther-University Halle, 06120 Halle, Germany

**Keywords:** Cancer, IGF2BP, IMP, CRD-BP, VICKZ, KOC, MYC, Migration, Proliferation

## Abstract

The insulin-like growth factor-2 mRNA-binding proteins 1, 2, and 3 (IGF2BP1, IGF2BP2, IGF2BP3) belong to a conserved family of RNA-binding, oncofetal proteins. Several studies have shown that these proteins act in various important aspects of cell function, such as cell polarization, migration, morphology, metabolism, proliferation and differentiation. In this review, we discuss the IGF2BP family’s role in cancer biology and how this correlates with their proposed functions during embryogenesis. IGF2BPs are mainly expressed in the embryo, in contrast with comparatively lower or negotiable levels in adult tissues. IGF2BP1 and IGF2BP3 have been found to be re-expressed in several aggressive cancer types. Control of IGF2BPs’ expression is not well understood; however, let-7 microRNAs, β-catenin (CTNNB1) and MYC have been proposed to be involved in their regulation. In contrast to many other RNA-binding proteins, IGF2BPs are almost exclusively observed in the cytoplasm where they associate with target mRNAs in cytoplasmic ribonucleoprotein complexes (mRNPs). During development, IGF2BPs are required for proper nerve cell migration and morphological development, presumably involving the control of cytoskeletal remodeling and dynamics, respectively. Likewise, IGF2BPs modulate cell polarization, adhesion and migration in tumor-derived cells. Moreover, they are highly associated with cancer metastasis and the expression of oncogenic factors (KRAS, MYC and MDR1). However, a pro-metastatic role of IGF2BPs remains controversial due to the lack of ‘classical’ in vivo studies. Nonetheless, IGF2BPs could provide valuable targets in cancer treatment with many of their in vivo roles to be fully elucidated.

**Electronic supplementary material:**

The online version of this article (doi:10.1007/s00018-012-1186-z) contains supplementary material, which is available to authorized users.

## Introduction

The insulin-like growth factor-2 mRNA-binding proteins 1, 2, and 3 (gene symbols: IGF2BP1, IGF2BP2, IGF2BP3) belong to a highly conserved protein family, which as their name suggests can bind RNA and influence their transcript target’s fate. Nomenclature of the IGF2BP protein family remains confusing due to the many synonyms used throughout recent literature including: IMP, CRD-BP, VICKZ, ZBP, Vg1RBP/Vera or KOC. These synonyms may reflect the evolution of the various fields of IGF2BP family research which suggest that these RNA-binding proteins (RBPs) modulate important aspects of cell function during development and in cancer. In this review, we discuss the rapidly growing research into the IGF2BP family’s involvement in cancer biology and the mechanisms by which high expression of these RBPs could cause an aggressive malignancy phenotype. We also discuss the molecular mechanisms by which these proteins facilitate their various functions, their role in cell migration and the need for better research tools to facilitate the next generation of IGF2BP research.

**Fig. 1:**
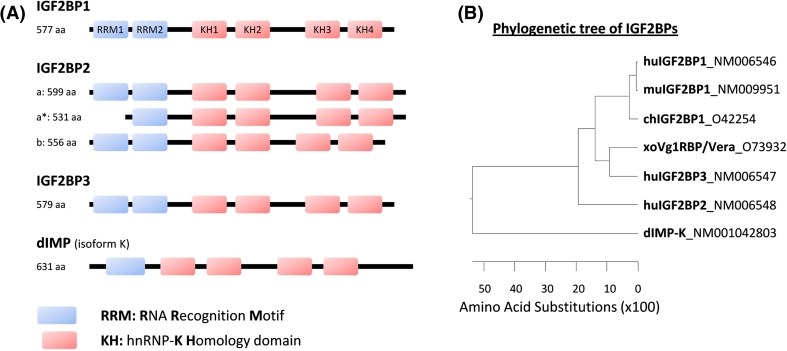
The IGF2BP protein family. **a** Domain structure of humanIGF2BPs and additionally, the IMP ortholog (*dIMP*, isoform K) of *Drosophila melanogaster*. RNA-binding domains comprising RNA recognition motifs (*RRM*s, *blue*) and hnRNP-K homology domains (*KH*, *red*). The following proteins are shown: *IGF2BP1* (Acc. no.: NM006546), the longest IGF2BP1 protein isoform; *IGF2BP2*-*a* (Acc. no.: NM006548), the longest IGF2BP2 protein isoform; *IGF2BP2*-a* (no Acc. no. available), truncated IGF2BP2-a resulting from leaky scanning during translation initiation [58]; *IGF2BP2*-*b* (Acc. no.: NM001007225.1), spliced IGF2BP-a lacking exon 10; *IGF2BP3* (Acc. no.: NM006547), the only reported variant of this paralogue; *dIMP* (Acc. no.: NM001042803), variant K of the *Drosophila melanogaster* ortholog of IGF2BPs. **b** Phylogentic tree indicating amino acid substitutions of distinct IGF2BP paralogues from different species (*hu* human, *mu* murine, *ch* chicken, *xo* *Xenopus*, *d* *Drosophila*). The accession number for each ortholog is indicated

In mammals, the canonical structures of the three IGF2BP proteins are strikingly similar in order and spacing of domains (Fig. 1a), leading to proteins of calculated molecular weights ranging from 58 to 66 kDa. There is over 56 % amino acid sequence identity between the three proteins with greater degree of similarity seen within the protein domains. These similarities suggest that the proteins share biochemical functions. Notably, IGF2BP1 and 3 show a higher identity of 73 % with each other (Fig. 1b). All three proteins carry two RNA-recognition motifs (RRMs) in their *N*-terminal part and four hnRNP-K homology (KH) domains in the *C*-terminal region. Notably, only one IGF2BP ortholog has been reported in *Xenopus*, termed Vg1RBP/Vera. This shows the highest homology to mammalian IGF2BP3. In *Drosophila*, a protein lacking the *N*-terminal RRM domain but comprising four KH-domains has been suggested as *Drosophila* IGF2BP (*dIMP*).

Regardless of organism or cell type, all members of the IGF2BP protein family have been shown to bind RNA, whereas an association with DNA has only been reported once for the *Xenopus* variant of protein [1]. In vitro studies revealed that RNA-binding is mainly facilitated via the KH-domains [2], although the RRM-domains could potentially contribute to the stabilization of IGF2BP-RNA complexes with target-dependent in vitro half-life greater than 2 h [3]. Recent structural analyses of human IGF2BP1 KH-domains 3 and 4 suggest the formation of an anti-parallel pseudo-dimer conformation in which KH3 and KH4 each contact the targeted RNA [4]. Although final proof of this hypothesis requires protein–RNA co-crystals, these findings suggest that IGF2BPs force associated transcripts into a specific conformation. In light of the surprisingly long half-life of IGF2BP-RNA complexes in vitro, this provides evidence for an essential role of IGF2BPs in promoting the formation of ‘stable’ protein–RNA complexes.

### The ribonucleoprotein (RNP) granule connection

IGF2BPs are predominately cytoplasmic, usually with a granular appearance. A nuclear role of IGF2BPs remains controversial, although there is evidence that IGF2BPs may already associate with their target mRNAs at their site of transcription [5–7]. In agreement, IGF2BPs were observed in the nucleus of spermatogenic cells and were suggested to comprise nuclear export signals [8]. In the cytoplasm, IGF2BPs form distinct ribonucleoprotein (RNP) granules which are enriched in the peri-nuclear region but are also observed in neurites of developing neurons supporting a role of IGF2BPs in promoting mRNA localization [2, 9]. Like most RNA-binding proteins (RBPs), IGF2BPs associate with various other RBPs in an RNA-dependent manner [10, 11]. However, in contrast to other proteins involved in the control of cytoplasmic mRNA fate, IGF2BPs apparently associate predominantly with ‘virgin’ mRNAs. This notion is supported by the observed association with components of the exon junction complex (EJC) as well as CBP80 whereas IGF2BPs do not copurify with eIF4E protein [10, 11]. Hence, IGF2BPs apparently ‘cage’ their target mRNAs in cytoplasmic protein–RNA complexes, termed mRNPs. This prevents the premature decay of specific target transcripts, for instance, CD44, MYC, PTEN or BTRC, presumably by limiting the release of protein-associated transcripts [12–16]. IGF2BP-directed recruitment of targeted mRNAs to cytoplasmic mRNPs is also consistent with their role in controlling mRNA translation and transport. The formation of stable protein–RNA association, as suggested based on in vitro studies [3], provides a bona fide mechanism to prevent promiscuous translation of transported mRNAs. The stable ‘caging’ of transported mRNAs allows for their ‘long-distance’ transport as well as transient storage. Consistently, IGF2BPs have been shown to direct the localization and spatially restrict translation of the β-actin (ACTB) mRNA to exploratory growth cones of developing neuronal cells [6, 9]. Moreover, IGF2BP1 was shown to stabilize its target transcripts during cellular stress when global mRNA translation is severely reduced and mRNAs together with RBPs are recruited to transiently forming stress granules [17].

**Fig. 2:**
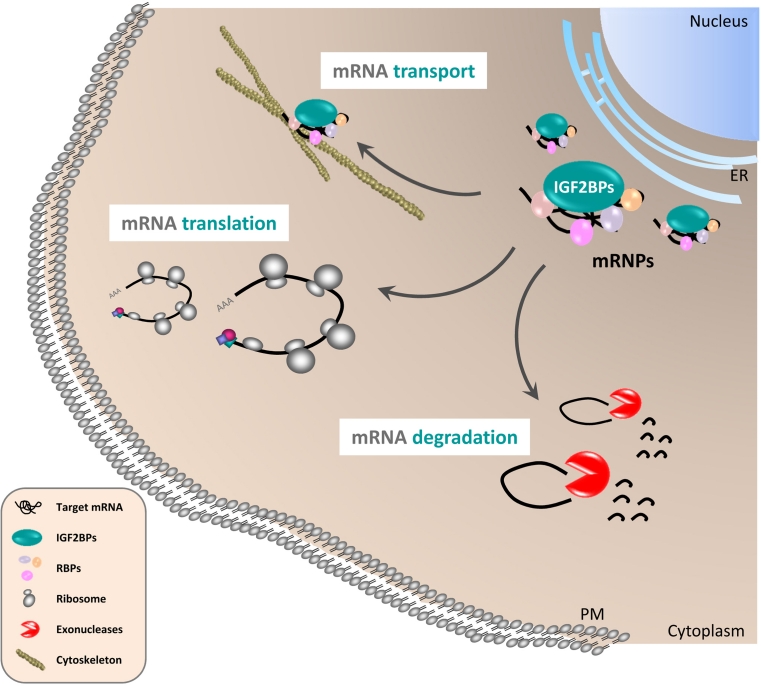
Regulation of cytoplasmic mRNA fate by IGF2BPs. IGF2BPs associate with specific target mRNAs and other RNA-binding proteins (RBPs) in cytoplasmic mRNPs. The release of associated mRNAs from these mRNPs results in either their decay (*mRNA degradation*) of or protein synthesis (*mRNA translation*). The formation of ‘stable’ mRNPs is presumed to allow the directed transport of specific mRNAs along the microtubule and/or actin cytoskeleton (*mRNA transport*). To prevent promiscuous translation of sorted mRNAs, localized transcripts are likely to be translationally silenced during transport

However, the efficient ‘caging’ of transcripts in cytoplasmic mRNPs requires signaling events allowing the controlled release of silenced mRNAs to induce protein synthesis or mRNA decay, respectively. In the case of IGF2BPs, this regulation is likely to involve phosphorylation of the proteins. Src-directed tyrosine phosphorylation in the linker region connecting KH-domains 2 and 3 of IGF2BP1 was proposed to induce the disassembly of cytoplasmic mRNPs and activate the translation of the ACTB mRNA [6]. Phosphorylation of Vg1RBP/Vera by MAPKs was suggested to modulate the release of Vg1 mRNA from mRNPs localized to the vegetal cortex during meiotic maturation [18]. Although not linked to mRNA localization, it was recently shown that phosphorylation of IGF2BP2 in the *N*-terminal linker region connecting RRM2 and KH1 by mTORC1 promotes the association with the leader3 5′-UTR of IGF2 resulting in elevated IGF2 protein synthesis [19]. Hence, the post-translational modifications of IGF2BPs emerge as an essential trigger modulating their role in controlling the cytoplasmic fate of specific transcripts. The underlying mechanism of these regulations would fit well with the idea that some target mRNAs of IGF2BPs are ‘caged’ in relatively stable cytoplasmic mRNPs (Fig. 2). However, why do we observe translational silencing of some target mRNAs whereas the association of IGF2BPs with other transcripts prevents their premature decay? Essentially, one could envision two mechanisms that are likely to cooperate in directing cytoplasmic mRNA fate. On the one hand, the protein composition of regulatory mRNPs could determine mRNA fate. Although this assumption remains largely speculative, transcript-specific mRNP compositions have been proposed [11]. Alternatively, final mRNA fate could be determined exclusively by cis-determinants of the regulated transcripts. In this scenario, the exclusive role of IGF2BPs would be to ensure the spatiotemporal execution of ‘final transcript fate’ by controlling the release of regulated transcripts from cytoplasmic mRNPs. Although not formally proven, this model is in agreement with various observations. For instance, IGF2BP1 was proposed to shield the BTRC (beta-transducin repeat containing E3 ubiquitin protein ligase) mRNA from microRNA-mediated degradation in the cytoplasm [20]. Likewise, IGF2BP1 was proposed to protect the MYC and MDR1 mRNAs from endonucleolytic attack [12, 21]. Moreover, it was proposed that the potential association of IGF2BPs with their target mRNAs already at the site of transcription provides an efficient mechanism to direct cytoplasmic mRNA fate by directing the assembly of mRNPs before cytoplasmic entry [5–7]. Consistently, IGF2BP1 was observed in ‘virgin’ mRNPs [10, 11]. Taken together, this suggests that IGF2BPs start controlling transcript fate right after transcription and modulate the rate at which associated transcripts encounter the translational apparatus, the decay machinery or microRNA attack by recruiting regulated transcripts in cytoplasmic mRNPs (Fig. 2). Although there is substantially more work required to clarify the molecular mechanisms by which IGF2BPs modulate mRNA fate, their role certainly involves cytoplasmic mRNPs and requires extensive control by cytoplasmic signaling.

### The ‘RNA-binding puzzle’ of IGF2BPs

Despite various studies indicating a specific role of IGF2BPs in controlling the localization, translation or turnover of specific mRNA targets (Table 1), a comprehensive identification of targeted transcripts is still lacking. PAR-CLIP and RIP studies have suggested more than 1,000 target mRNAs for IGF2BP1 [10, 22]. However, it should be noted that these studies were based on the stable expression of Flag-tagged proteins in HEK293 cells, in which the stable expression of IGF2BP1 results in aberrant sedimentation in polysomal gradient centrifugation when compared with endogenous protein (Fig. S1). Recent studies focusing on structural constrains defined by the KH-domains 3 and 4 suggested just over 100 mRNAs to be regulated by IGF2BPs [23]. However, these studies do not take into account that KH-domains 1 and 2 are likely to be involved in RNA-binding; also, as the studies were based on IGF2BP1, the repertoire for the entire IGF2BP family could be significantly larger. A role of KH-1/2 in RNA-binding is supported for instance by the finding that in vitro KH3/4 do not associate with RNA below concentrations of 100 nM, unlike the full length protein [3]. Moreover, we observed that the KH1/2 domain modulates binding of IGF2BP1 to cis-determinants in the ACTB 3′UTR and, more strikingly, the MYC-CRD (coding region stability determinant) RNA in vitro (Fig. S2). This could indicate that KH1/2 are important for the stabilization of IGF2BP-RNA complexes.

**Table 1:** Target mRNAs of IGF2BPs

Target	Cis-element on RNA	IGF2BP	Regulation of target mRNA	References
ACTB	3′-UTR	1	Inhibition of mRNA translation	[6, 14, 43, 44]
ACTB	3′-UTR	1	mRNA transport	[2, 9, 42, 84]
BTRC	CDS	1	Inhibition of miR-dependent mRNA decay	[16, 20]
CD44	3′-UTR	1, 3	Inhibition of mRNA decay	[15]
CTNNB1	3′-UTR	1	Inhibition of mRNA decay	[50]
GLI1	Nd	1	Inhibition of mRNA decay	[98]
*Gurken*	5′-UTR	dIMP	mRNA transport/translation	[34]
IGF2	5′-UTR	1	Inhibition of mRNA translation	[31]
IGF2	5′-UTR	2, 3	Enhancement of mRNA translation	[19, 70–72]
MAPK4	3′-UTR	1	Inhibition of mRNA translation	[14]
MDR1	CDS	1	Inhibition of CRD-dependent mRNA decay	[21]
MYC	CDS	1	Inhibition of CRD-dependent mRNA decay	[11–13, 65, 66]
*Oskar*	3′-UTR	dIMP	mRNA transport/translation	[33]
PPP1R9B	3′-UTR	1	mRNA transport	[23]
PTEN	CDS	1	Inhibition of CRD-dependent mRNA decay	[14]
Vg1	3′UTR	Vg1RBP/Vera	mRNA transport/translation	[99–101]
*HCV*	5′-/3′-UTR	1	Enhancement of translation	[102]

Target	Cis-RNA	IGF2BPs	Proposed regulation of target RNA	References
CDH1	–	1	mRNA localization	[103]
H19	ncRNA (+)	1, (3)	mRNA localization, IGF2 expression	[3, 104]
LAMB2	–	2	Control of mRNA translation	[89]
LIMS2	–	2	Inhibition of mRNA decay	[90]
KRAS	CDS, 3′-UTR	1	Inhibition of mRNA decay	[57]
MAPT	–	1	mRNA localization	[105, 106]
PABPC1	5′-UTR	1	mRNA translation	[107]
PTGS2	–	1	mRNA increase (undefined)	[91]
TRIM54	–	2	Inhibition of mRNA decay	[90]
Y3	ncRNA(+)	1, 2, 3	RO60 protein localization	[97, 108]

Taken together, the currently available studies suggest a significant structural complexity of IGF2BP-RNA association. Structural studies of KH3/4, although still lacking protein–RNA co-crystal information, suggest that each KH-domain of IGF2BPs, presumably including KH-domains 1 and 2, forms direct contacts with associated transcripts [4]. Assuming that PAR-CLIP identifies specific binding consensus motifs, a putative binding motif for the KH-domains of IGF2BPs could be CAUH (H = A, U, or C) [22]. Thus, only the defined spacing of specific association motifs on substrate RNAs would determine the formation of specific IGF2BP–RNA complexes in vivo. Another layer of complexity to be considered is that IGF2BPs form homo- and potentially hetero-dimers on their target mRNAs and that this was proposed to promote the formation of stable protein–RNA complexes [3, 24]. In agreement, the stability of IGF2BP–RNA complexes was found to increase with the length of probed RNA baits in vitro whereas K_D_-values were decreased [3]. Hence, it appears as if the identification of physiological relevant target mRNAs of IGF2BPs cannot be based solely on studying protein–RNA association, but presumably requires functional screening approaches and correlation with cellular functions of the IGF2BP protein family.

### The role of IGF2BPs during development

**Fig. 3:**
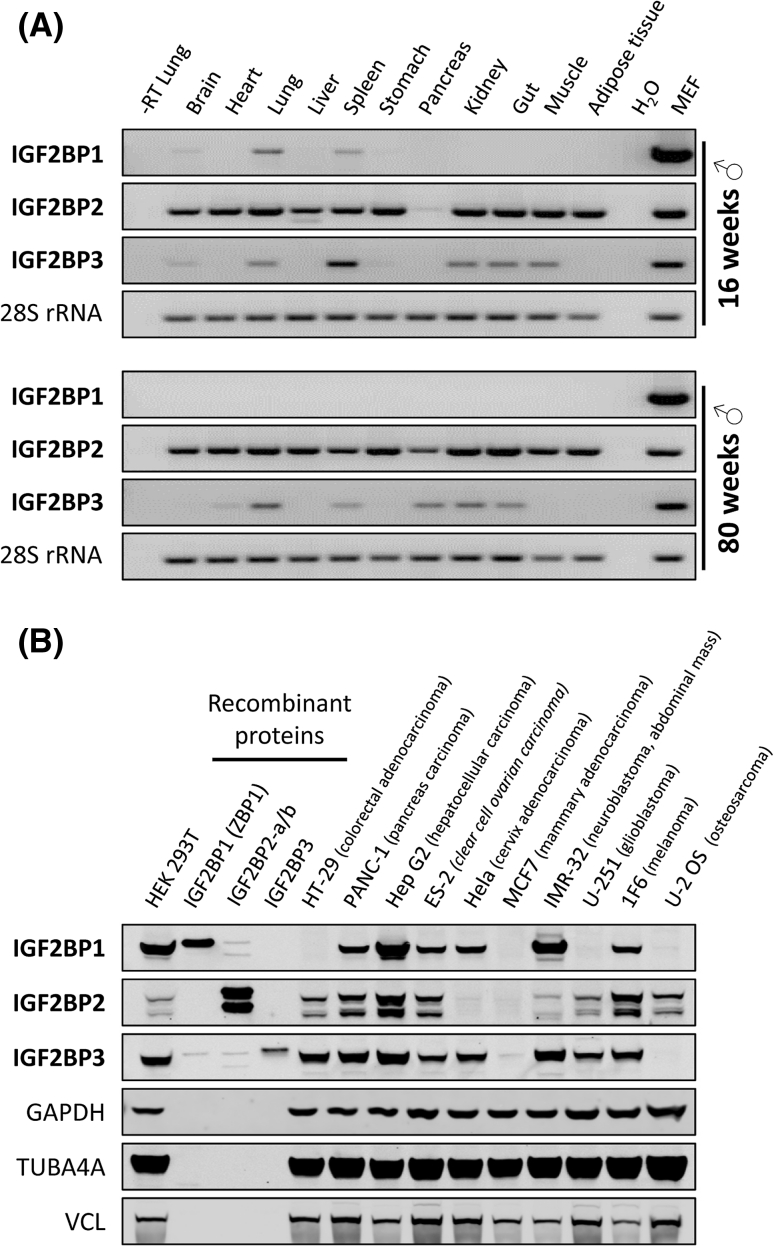
IGF2BP expression in adult mice and tumor-derived cells. **a** Semi-quantitative RT-PCR analysis of IGF2BP expression (40 PCR cycles) in adult mouse tissues. Total RNA was analyzed from tissues isolated from either a 16- or 80-week-old male mouse. 28S RNA served as a loading control (20 PCR cycles). Total RNA isolated from E17 mouse embryonic fibroblasts (*MEF*) was used as positive control. Total lung RNA without reverse transcription (−RT) and water served as negative controls. **b** IGF2BP protein expression in indicated tumor-derived cells was analyzed by western blotting using mouse monoclonal antibodies directed against each of the three paralogues. Recombinant IGF2BP proteins (20 ng; including IGF2BP2-a and IGF2BP2-b) served as controls. Note, the IGF2BP3-directed antibody shows a significant cross-reactivity with IGF2BP1 (see also supplemental Fig. S4), presumably reflecting the high sequence similarity of both proteins. The cross-reactivity of both anti-IGF2BP1 (6A9) and anti-IGF2BP3 (6G8) with IGF2BP2 is low and presumably negligible for most studies (see also supplemental Fig. S4). Notably, one or two IGF2BP paralogues are expressed at very low levels in some tumor-derived cells, whereas all three paralogues are expressed in other cancer-derived cells. Additional controls for paralogue specificity of used monoclonal antibodies are shown in Fig. S4

An important characteristic of the IGF2BP family is its high expression during the period between zygote and embryo stages [25]. There is a sharp peak in expression seen around embryonic day 12.5 before a decline in expression towards birth in mice [25, 26]. At E12.5, IGF2BPs are expressed at very high levels in the brain, limb buds, and muscle, and in the epithelia of many organs in mice. During *Xenopus* development, Vg1RBP/Vera is also expressed in the neural tube and neural crest cells [27]. Compared to their high expression in the embryo, IGF2BP1 and IGF2BP3 were reported to be expressed at negligible levels in adult organs, with the exception of reproductive tissues [26]. In contrast, IGF2BP2 was suggested to be expressed in various adult tissues (reviewed in [28–30]). Aiming to re-evaluate these observations, we analyzed the expression of IGF2BPs in various adult mouse tissues by semi-quantitative RT-PCR (Fig. 3a). These studies confirmed that IGF2BP1 expression is essentially abolished in the adult organism, although modest expression was observed in the brain, lung and spleen of 16-week-old male mice. Largely age-independent although modest expression of IGF2BP3 was observed in the lung, spleen, kidney, and gut of male mice. Surprisingly, expression in the brain and muscle was only observed in 16-week-old mice, whereas modest expression was observed in the heart and pancreas of 80-week-old mice. Consistent with previous reports, largely age-independent expression of IGF2BP2 was observed in all analyzed tissues, except pancreas. In the latter, IGF2BP2 expression appeared to be upregulated in 80-week-old mice. All family members were expressed in E17 mouse embryonic fibroblasts (MEFs). Hence, the expression pattern observed for IGF2BP1 and IGF2BP3 can indeed be characterized as ‘oncofetal’, since they are largely absent from adult tissues but de novo synthesized or severely upregulated in various tumors and tumor-derived cells (Fig. 3b; reviewed in [28, 29]). In contrast, IGF2BP2 seems to be the only family member involved in directing mRNA fate in non-transformed adult tissues, supporting a role for this protein in metabolic control (reviewed in [30]).

The only family member for which knockout mice have been reported is IGF2BP1. Mice deficient for this family member have severely reduced viability, dwarfism and impaired gut development [25]. The smaller sized organs and 40 % smaller sized animals were suspected to be caused via hypoplasia. PCNA, a marker of proliferating cells, was reduced and a marker of apoptosis (TUNEL staining) was not significantly increased compared to wild-type mice. This indicates a pivotal role of IGF2BP1 in promoting cell growth and differentiation during development, presumably involving the regulation of IGF2 mRNA translation [31].

In *Drosophila,* loss of function *dIMP* mutations are zygotic lethal and the overexpression of dIMP disrupts dorsal/ventral polarity [32]. Consistently, *dIMP* could possibly direct the fate of localized mRNAs during early development, including gurken and oskar [33, 34]. As observed in vertebrates, *dIMP* shows a biphasic expression during embryogenesis and is expressed in reproductive tissues [35–37]. Moreover, *dIMP* plays a role in determining cell fate in testis stem cells and modulates neuronal differentiation [32, 38]. Hence, in all organisms analyzed so far, IGF2BPs were identified as essential modulators of cell growth and differentiation during development. IGF2BP1 and IGF2BP3 can be considered ‘oncofetal’ proteins with a biphasic expression during development and significant upregulation in various malignancies (see Tables 2, 3). Consistent with a suggested role in metabolic control, the only family member widely expressed in adult mouse tissues is IGF2BP2.

**Table 2:** IGF2BP1 expression in human cancers

Cancer	Method	Incidence	References
Breast	RT-PCR	59 % (69/118)	[109]
Ovarian carcinomas	IHC	69 % (73/106)	[13]
Ovarian	IHC	Not done (associated with MDR1)	[56]
Testis	IHC	90 % (30/33)	[26]
Brain tumors (various)	RT-PCR	55 % (28/51)	[110]
Melanoma	IHC	34 % (13/38)	[111]
Non-small cell lung	RT-PCR	27 % (4/11)	[110]
Pancreatic	Northern	33 % (5/15)	[112]
Colon, lung, ovarian	IHC	>60 %	[61]
Colon	IHC, RT-qPCR	50 % (36/78), 59 % (46/78)	[52]
Colorectal	RT-PCR	81 % (17/21)	[51]
Mesenchymal	RT-PCR	65 % (28/43)	[113]
Hodgkin lymphoma	IHC	94 % (101/108)	[78]
B cell lymphomas (various)	IHC	69 % (458/661)	[78]

**Table 3:** IGF2BP3 expression in human cancers

Cancer	Incidence (%)	References
Gastrointestinal/pancreatic		
Pancreatic adenocarcinoma	63–97	[114–119]
Esophageal adenocarcinoma	66–94	[120, 121]
Gastric adenocarcinoma	60	[122]
Colorectal adenocarcinoma	65–74	[123, 124]
Hepatobiliary		
Hepatocellular carcinoma	53–68	[79, 125]
Bile duct carcinoma	58	[126]
Gynecologic		
Endometrial clear cell carcinoma	39	[74]
Endometrioid carcinoma	7–46	[74, 127]
Serous endometrial carcinoma	94–100	[59, 74, 127]
Cervical adenocarcinoma in situ	21–93	[75, 128]
Ovarian carcinoma	47	[76, 129]
Lung/pleura		
Non–small cell lung cancer	55	[130]
Squamous cell carcinoma lung	75–90	[130, 131]
Adenocarcinoma of the lung	70–90	[131, 132]
Bronchioloalveolar carcinoma	25–40	[130, 132]
Malignant mesothelioma	36–91	[133, 134]
Lymphoid		
Hodgkin lymphoma	100	[77]
Burkitt lymphoma	83	[77]
Follicular lymphoma	80	[77]
Diffuse large B cell lymphoma	85	[77]
Cutaneous		
Melanoma	40–50	[80, 135]
Merkel cell carcinoma	90	[136, 137]
Thyroid		
Papillary carcinoma, conventional	11–87	[138, 139]
Papillary carcinoma, follicular variant	38–67	[138, 139]
Follicular carcinoma	63–69	[138, 139]
Hürthle cell carcinoma	21	[138]
Poorly differentiated carcinoma	59	[140]
Nervous system		
Meningioma	6.5	[141]
Pituitary adenoma	31	[142]
Pituitary carcinoma	36	[142]
Neuroblastoma	58	[81]
Genitourinary		
Renal cell carcinoma, overall	11–21	[143, 144]
Renal cell carcinoma, clear cell	14–30	[143, 144]
Renal cell carcinoma, chromophobe	15–35	[143–145]
Renal cell carcinoma, papillary	9–65	[143–145]
Noninvasive papillary urothelial carcinoma	1–53	[146]
Urothelial carcinoma in situ	36–48	[146, 147]
Invasive urothelial carcinoma	34–59	[146, 147]
Breast		
Mammary carcinoma	33–41	[148–150]
Other		
Extrapulmonary small cell carcinoma	94	[151]
Mesothelioma	73	[152]
Osteosarcoma	17–96	[153, 154]

### The role of IGF2BPs in the nervous system

The spatiotemporal control of mRNA localization is considered a key determinant of neuronal development, cytoskeletal remodeling, and finally synaptic function (reviewed in [39, 40]). IGF2BPs were identified as key players in these processes due to their role in directing subcellular mRNA sorting and spatial control of key mRNA translation. A few transcripts have been suggested to be regulated in a spatiotemporal manner by IGF2BPs in neurons (Table 1). However, the role of IGF2BP1 in controlling the fate of the ACTB mRNA is the most investigated (reviewed in [41]). The current view suggests that IGF2BP1 promotes the assembly of relatively stable cytoplasmic mRNPs comprising the ACTB mRNA. This allows the directed transport of the translationally silenced transcript into developing axons and dendrites [9, 42]. Spatially restricted translation of localized ACTB mRNAs is presumably activated by Src-mediated phosphorylation of IGF2BP1 [6]. This spatiotemporal fine tuning of ACTB protein synthesis was suggested to promote growth cone guidance during development [43–45]. Recent studies indicate that IGF2BP1 also promotes the outgrowth and branching of neurites in hippocampal neurons, presumably by controlling Src-dependent spatiotemporal activation of ACTB protein synthesis [46]. Notably, these studies revealed that IGF2BP1 is not required for the maintenance of matured dendrites, correlating well with the observation that IGF2BP1 is not expressed in the adult mouse brain, although final proof of this assumption requires further in depth analyses (Fig. 3a). Notably, IGF2BP1 was recently implicated in nerve regeneration capacity of adult sensory neurons, suggesting that the protein could also play a role in the matured neuronal system, at least during regeneration [47]. Studies in *Drosophila* and *Xenopus* support essential roles of IGF2BPs in the nervous system. In *Drosophila,* *dIMP* was revealed to promote synaptic terminal growth and modulate protein synthesis at neuromuscular junctions [32]. In *Xenopus*, the ortholog Vg1RBP/Vera was shown to be required for migration of cells forming the neural tube of the embryo and, subsequently, migration of neural crest cells [27]. Taken together, these findings identify IGF2BPs as key regulators of neuronal development that modulate neurite outgrowth and neuronal cell migration, presumably by the spatiotemporal fine tuning of protein synthesis, as demonstrated for ACTB.

### Control of IGF2BP expression

Surprisingly little is known about how the expression of IGF2BPs is regulated at the transcriptional level. In HEK293 cells, IGF2BP1 transcription was proposed to be induced by β-catenin (CTNNB1) in a TCF-dependent manner [16]. This observation remains puzzling, since the authors propose that, without CTNNB1/TCF4 overexpression, IGF2BP1 mRNA is not present or barely observed in HEK293 cells. In contrast, various studies indicate that IGF2BP1 is highly abundant in HEK293 cells (e.g., [6, 10, 48]). Despite this controversy, the CTNNB1-induced activation of IGF2BP1 expression was proposed to promote IGF2BP1-dependent stabilization of the BTRC and MYC mRNAs leading to elevated expression of both proteins [16]. While IGF2BP1 stabilizes the MYC mRNA presumably by protecting the transcript from endonucleolytic attack, the protein was proposed to prevent miR-182 directed degradation of the BTRC transcript [12, 13, 20]. These observations suggest that IGF2BP1 transcription is modulated by negative as well as positive feed-back regulation. Negative feed-back regulation should be facilitated by BTRC-dependent degradation of CTNNB1, whereas MYC was proposed to enhance the transcription of IGF2BP1, suggesting a positive feed-back loop [49]. Controversially, CTNNB1 was proposed to enhance the expression of IGF2BP1 expression by positive feed-back regulation in mammary carcinoma-derived tumor cells [50]. Taken together, the presented studies support the view of an oncogenic role of IGF2BP1 by providing evidence for CTNNB1/TCF4 as well as MYC-dependent transcriptional activation. This is consistent with the severe upregulation of IGF2BP1 in various malignancies (Table 2) and correlates well with IGF2BP1 de novo synthesis observed in colorectal carcinomas [51, 52]. However, substantially more work is required to decipher the cross-talk and feed-back regulations which are likely to orchestrate IGF2BP1 transcription in a cell- and malignancy-dependent manner.

Little information is available on the transcriptional control of other IGF2BPs. Transcriptional regulation of IGF2BP3 has never been studied to our knowledge. Two studies indicate that IGF2BP2, but not the two other family members, is regulated by the ‘architectural’ transcription factor HMGA2 and NFκB (NFKB1). The first report on the control of IGF2BP2 expression convincingly demonstrates that transcription of this paralogue is essentially abolished in HMGA2 (-/-) mice [53]. Consistently, HMGA2 was later proposed to promote the transcription of IGF2BP2 by associating with an AT-rich region in the first intron of the IGF2BP2 gene [54]. Remarkably, the same region is targeted by NFKB1 that apparently synergizes with HMGA2 in enhancing the transcription of IGF2BP2. Hence, in contrast to IGF2BP1 where transcriptional control is proposed to be orchestrated via a bona fide promoter region located upstream of the start codon, IGF2BP2 expression is suggested to involve enhancer elements located in the first IGF2BP2 intron.

The post-transcriptional control of mRNA fate is a main regulatory crank in the control of gene expression. In this respect, a study by the Bartel laboratory provided a new perspective that emphasizes the 3′-end of IGF2BP transcripts, in particular IGF2BP1, in modulating the expression of this gene family [48]. Consistent with various in silico-predicted poly-adenylation sites in the approximately 7-kb-long 3′-UTR of the transcript (Fig. S3), at least three IGF2BP1 transcripts were observed in various tumor-derived cells and HEK293 cells. This supports the notion that IGF2BP1 expression is modulated by alternative poly-adenylation (APA). Although the mechanism by which APA of IGF2BP1 is controlled remains largely elusive, it is commonly accepted that 3′-UTR shortening provides a potent escape strategy preventing the targeting of repressive microRNAs. This appears to be preferentially observed for transcripts encoding oncogenic factors which are targeted by tumor-suppressive microRNAs like the let-7 family, as demonstrated for IGF2BP1 [55]. Notably, APA-sites are only suggested for IGF2BP1 based on currently available sequence information (Fig. S3). Whether this indicates that 3′-UTR shortening provides an escape strategy only for IGF2BP1 remains to be elucidated.

The observed post-transcriptional control of IGF2BP1 expression by microRNAs was suggested to modulate tumor cell fate. Downregulation of let-7 expression, frequently observed in aggressive tumor cells, was correlated with increased drug-resistance and an upregulation of IGF2BP1 [56]. The latter was proposed to enhance the expression of the multi-drug-resistance factor 1 (MDR1) by preventing MDR1 mRNA degradation via endonucleases, as previously proposed for MYC [21]. Hence, the microRNA-dependent upregulation of IGF2BP1 enhanced drug resistance by promoting the expression of MDR1. This supports other studies which indicate that regulatory post-transcriptional networks modulate tumor cell properties. For IGF2BP1, it was demonstrated that the protein promotes the expression of various bona fide let-7 targets including KRAS, Lin-28B and MYC [57]. Notably, the role of IGF2BP1 in the let-7-dependent post-transcriptional control of gene expression is apparently conserved through evolution. In *Drosophila*, let-7-controlled expression of *dIMP* was recently proposed to modulate the expression of the self-renewal factor Upd in the testis stem cell niche [38].

Although regulation of the other IGF2BP family members by microRNAs has not so far been demonstrated, the expression of at least IGF2BP2 seems to be also regulated at the post-transcriptional level. Recent studies indicate that leaky scanning during translation initiation results in the expression of a shorter protein isoform [58]. We have confirmed the expression of this isoform in osteosarcoma-derived U2OS cells and demonstrated that at least three protein isoforms of IGF2BP2 are expressed in several tumor-derived and transformed cells (Fig. 3b; Fig. S4). These include the longest protein isoform (IGF2BP2-a; Acc. no.: NM006548.4; calculated MW: 66 kDa), an alternatively spliced variant lacking exon 10 (IGF2BP2-b; Acc. no.: NM001007225.1; calculated MW: 61.8 kDa) and presumably the shortest isoform resulting from leaky scanning of IGF2BP2-a with a calculated molecular weight of 58.6 kDa (IGF2BP2-a*). As for IGF2BP2, an alternative splice variant lacking exons 6 and 7 was proposed for IGF2BP1 (Acc. no.: NM 001160423.1). However, although we were able to generate a cDNA encoding the shorter IGF2BP1 isoform by RT-PCR cloning from HEK293 cells, we have not been able to conclusively demonstrate expression of the shorter protein variant at the endogenous level (data not shown).

Taken together, it remains poorly understood how the transcription of IGF2BPs is regulated and how it might be modulated by epigenetic mechanisms. In contrast, there is substantial evidence for a significant role of post-transcriptional mechanisms directing the control of at least IGF2BP1 expression. The ‘let-7-axis’ appears to emerge as a highly conserved regulatory mechanism that antagonizes the expression of IGF2BP1. This supports the view that IGF2BP1 enhances tumor cell aggressiveness, since the let-7 microRNA family is considered to facilitate a tumor-suppressive role in most malignancies. Nonetheless, substantial efforts are required to promote our understanding of how the expression of IGF2BPs is modulated by the interplay of transcriptional and post-transcriptional networks. This will provide essential insights into how IGF2BP function is controlled during development and becomes deregulated in diseases.

### Expression of IGF2BPs in cancer

Expression of IGF2BP family members has been implicated in various cancers; however, the vast majority of reports consider exclusively IGF2BP1 and IGF2BP3 (Tables 2, 3). For the latter, the most cited malignancies are those of the colon, liver, kidney, pancreas, and female reproductive tissues. There is sparse and less convincing evidence thus far for an oncogenic role for IGF2BP2, but studies have correlated the expression of this paralogue with liposarcoma, liver cancer, and endometrial adenocarcinomas [54, 59, 60]. This is consistent with the observation that IGF2BP1 and to a lesser extent also IGF2BP3 are mainly or even exclusively expressed during embryogenesis but become de novo synthesized in various malignancies. In contrast, IGF2BP2, which has barely been associated with a role in cancer, is the only paralogue observed to be expressed in all non-transformed mouse tissues so far analyzed (Fig. 3a).

The reported expression of IGF2BP1 and IGF2BP3 in primary malignancies does not allow concluding a specific expression pattern discriminating both paralogues. However, it should be noted that IGF2BP1 expression has been studied largely on the mRNA level by RT-PCR, whereas IGF2BP3 expression was analyzed mainly by immunohistochemistry. The latter is problematic with IGF2BPs due to the high sequence identity and homology. This imposes the difficulty to raise paralogue-specific antibodies which are useful for immunohistochemistry. Thus, isoform-specific expression analyses should be evaluated with caution and we expect that at least some of the reported observations have to be reconsidered.

### IGF2BP1—oncogene(ic) or not?

For the majority of studies, there is a severe gap between pure functional in vitro studies and more descriptive clinical oncology/epidemiology studies. For example, even though there is a large body of in vitro evidence for IGF2BP1 in promoting cell movement, the significance of IGF2BP1 in the process of cancer metastasis has not been directly confirmed through in vivo studies. Likewise, we still have little information on a putative co-regulation of IGF2BP1 and target mRNA expression in primary tumor samples, although the expression of IGF2BP1 has, for instance, been correlated with lymph node metastasis of colorectal carcinomas [61].

Only one study provides strong in vivo evidence for a pro-oncogenic role of IGF2BP1 by applying classical methods. In a transgenic mouse model, the expression of IGF2BP1 was induced in mammary epithelial cells of adult female mice via the whey acidic promoter (WAP) upon lactation [62]. The incidence of mammary tumors within 60 weeks was 95 % when IGF2BP1 was highly expressed, and still reached 60 % with lower relative expression of the paralogue. Tumors were generally multifocal and several tumor-bearing mice had metastases. The quantification of IGF2BP1 target RNAs demonstrated that levels of ACTB and MYC transcripts were unaffected by IGF2BP1 overexpression, whereas IGF2 and H19 were significantly and consistently induced at the RNA level in mammary tissue of transgenic mice after lactation. These findings are surprising for two reasons. In vitro, IGF2BP1 was shown to enhance the expression of MYC by preventing MYC mRNA degradation, whereas this was not observed in vivo, at least in the WAP-dependent mouse model [12, 13, 62]. Moreover, in vitro evidence indicates a role of IGF2BPs in modulating the translation of the IGF2 mRNA, mainly by associating with one of four known 5′-UTRs of IGF2, whereas total IGF2 mRNA levels were upregulated in vivo [31, 62]. In vitro studies revealed that IGF2BP1 also binds to the 3′-UTR of IGF2 mRNA which is identical in all IGF2 transcript variants. This could indicate that IGF2BP1 simply prevents IGF2 mRNA degradation in vivo [3]. Alternatively, one could envision a role of IGF2BP1 in modulating the activation and/or imprinting of the IGF2-H19 tandem locus (reviewed in [63]). Of note, the H19 RNA was reported to encode at least one microRNA, proposed to negatively affect cell proliferation, which would be consistent with the role of H19 as a tumor suppressor (reviewed in [64]). How this correlates with the observed induction of primary lesions as well as metastases in WAP-driven IGF2BP1 mouse models remains to be addressed [62].

IGF2BPs could be exploited in cancer through their influence on classical oncogenes, in particular MYC and KRAS [57]. Unlike various other targets to which IGF2BP1 binds via the 3′-UTR, IGF2BP1 was proposed to bind to the CRD in the MYC open reading frame [65]. There is a bulk of evidence accumulated indicating that IGF2BP1 sustains MYC expression in tumor cells derived from various cancers in vitro (e.g.: mammary carcinomas [66]; ovarian carcinomas [13]; colorectal carcinomas [57]). This regulatory role was mainly correlated with the role of IGF2BP1 in preventing cleavage of the MYC mRNA by endonucleases upon the stalling of ribosomes in a rare codon stretch at the 5′-end of the CRD [11, 12, 21, 67]. However, in light of the reported repression of MYC as well as KRAS expression by the let-7 microRNA family, which targets in the 3′-UTR of both transcripts, one could envision that IGF2BP1 also prevents the targeting of this miR-family. In the case of MYC, this could either be facilitated by blocking let-7 targeting to the MYC-3′-UTR or by recruiting the mRNA into cytoplasmic mRNPs upon association with the MYC-CRD. Alternatively, or in addition, the protein could prevent the targeting of miRs to the MYC-CRD, as previously proposed for the IGF2BP1-directed stabilization of the BTRC mRNA [20]. Evidence for an IGF2BP1-dependent enhancement of KRAS expression is presented by only one study, but the molecular mechanism of this regulation remains elusive [57]. However, the fact that both MYC and KRAS are targeted by microRNAs of the let-7 family, like IGF2BP1 itself, suggests that IGF2BP1 could prevent targeting of KRAS by this microRNA family.

Taken together, there is strong evidence for an ‘oncogenic’ role of at least IGF2BP1. However, there are obvious discrepancies between in vitro and the only available in vivo study. Hence, substantial efforts using in vivo models are required to elucidate the role of IGF2BPs in cancer.

### What is the role of IGF2BP3 in cancer?

In contrast to IGF2BP1, which has been extensively studied in vitro, the role of IGF2BP3 remains barely investigated. However, of the three family members, IGF2BP3 has been associated the most with distinct cancer types. Accordingly, it was suggested as an important biomarker in systemic malignancies (reviewed in [68, 69]).

Functional studies addressing a regulatory role of IGF2BP3 revealed essentially two validated target mRNAs and some putative candidates. Evidence indicating IGF2BP3 to promote the mRNA translation of leader3 IGF2 mRNAs was presented by two laboratories [70, 71]. These studies suggest that the protein, like IGF2BP2 [19], enhances the translation of IGF2 mRNAs carrying a highly structured 5′-UTR, the so-called leader3. The latter presents one out of four distinct 5′-UTRs encoded by the human IGF2 locus. In agreement, it was demonstrated that IGF2BP3 promotes cell growth, proliferation, and resistance to ionic irradiation in an IGF2-dependent manner [72]. In contrast, IGF2BP1 was proposed to repress the translation of the IGF2 mRNA, either via the leader3 5′-UTR or potentially via the 3′-UTR of the IGF2 mRNA [3, 31]. Although the IGF2BP paralogue-specific regulation of IGF2 expression might well be regulated in a cell type- or cancer progression-dependent manner, these and various other findings indicate IGF2 as a key target transcript of the IGF2BP protein family. Interestingly, however, IGF2BP3 was also correlated with increased in vitro invasiveness and metastasis in *Xenograft* studies [15, 71, 73]. The only validated target mRNA which provides a conclusive hint how IGF2BP3 could facilitate a pro-invasive role is CD44. Together with IGF2BP1, IGF2BP3 was shown to enhance the formation of invadopodia by preventing the degradation of the CD44 mRNA upon associating with the 3′-UTR of the CD44 mRNA [15].

In light of the poorly understood role of IGF2BP3 in modulating tumor cell functions, it is surprising to observe that there was an ‘explosion’ of descriptive studies published from 2007 onwards, which suggest IGF2BP3 expression to correlate with tumor aggressiveness in a broad variety of malignancies (Table 3). Among the various cancers for which an upregulation or de novo synthesis for IGF2BP3 was reported, lung, gastrointestinal, and ovarian cancers are the most frequently reported. Overall, in gastrointestinal cancers, there is the suggestion that IGF2BP3 expression, almost exclusively analyzed on the basis of immunostaining, correlates with an overall poor prognosis, tumor aggressiveness, and metastasis (for references, please refer to Table 3). In cancers of female tissues, positive staining was reported in 94 % of all serous endometrial carcinomas and 89 % of all serous endometrial intraepithelial carcinoma [74]. Notably, no expression was observed in endometrial intraepithelial neoplasia, whereas significant expression was observed in 93 % of cervical adenocarcinomas [75]. Notably, there is contradictive evidence for ovarian cancer suggesting IGF2BP3 expression to correlate with an improved survival [76]. One descriptive study by King et al. [77] displayed striking images of high IGF2BP3 protein expression by IHC in normal germinal centers of lymph nodes and negative staining in the periphery of the lymph nodes. Of note, a similar pattern was observed for IGF2BP1 in another lymphoma study [78]. The research of King and colleagues could support a role of IGF2BP3 in the proliferation and differentiation of B cells and possibly hints towards a broader role for IGF2BP3 in unrestricted proliferation and cell survival. Aside from these data, they also demonstrate a possible association of IGF2BP3 expression in specific subsets of lymphoma, such as 100 % of Hodgkin lymphoma. Although displaying less convincing IGF2BP3 staining in liver cancer, IGF2BP3 expression was correlated with cell proliferation by co-expression of ki67 [79]. This paralogue has also been associated with two cell types of neural crest origin; neuroblastoma and melanoma. IGF2BP3 has been found to be significantly highly expressed in metastatic melanomas, compared with thin melanomas. Thus, this paralogue may be useful diagnostically as a marker to differentiate melanoma from benign nevi cell types characterized by little or no IGF2BP3 expression [80]. Of relevance here is that Vg1RBP/Vera, the *Xenopus* ortholog of IGF2BPs, was revealed to promote the migration of neural crest cells during development [27]. This could indicate a significant role of IGF2BPs in the etiology of neuroblastoma and melanoma. In agreement, IGF2BP3 was proposed a marker of high clinical significance in neuroblastoma, with IGF2BP3-positive patients having decreased overall survival [81]. Interestingly, retinoic acid treatment of neuroblastoma cells revealed downregulation of IGF2BP3, and evidence within our laboratory shows this is also the case for IGF2BP1 (Bell et al., unpublished). Retinoid treatment causes the vast majority of neuroblastoma-derived cells to differentiate, decreases proliferation, and is therefore used in treating minimal residual disease neuroblastoma patients, but notably is also beneficial in many other cancers and proliferative disorders [82]. This could further implicate that high expression of IGF2BPs is associated with a de-differentiated highly proliferative cell state and speculatively nuclear receptor signaling pathways.

Taken together, evidence for an ‘oncogenic’ role of IGF2BP3 provided by in vitro studies is sparse and the paralogue specificity of used antibodies remains to be validated. Nonetheless, the bulk of correlative studies associating the upregulation of IGF2BP3 with various malignancies provide strong evidence for a pivotal role of IGF2BP3 in cancer.

### IGF2BPs as pro-survival factors

Obviously, the ability of IGF2BPs to increase the expression of MYC, IGF2 and potentially other pro-survival proteins like KRAS tends towards IGF2BPs themselves having pro-survival traits. This is a major characteristic of both oncogenes and embryonic growth factors and thus supports the oncofetal expression of IGF2BP1 and IGF2BP3. Recent studies have suggested both these paralogues to promote cell survival in response to Taxanes treatment or ionizing radiation, respectively [56, 72]. Both articles discuss common treatment regimens in cancer therapy imposing cell cycle arrest and/or apoptosis. Thus, the pro-survival role of IGF2BP1 and IGF2BP3 in response to these therapeutic treatments in vitro suggests that IGF2BPs also serve a role in mediating chemo-/radio-resistance of tumor cells. In support of this view, IGF2BP1 was shown to enhance the expression of MDR1 [21]. Notably, IGF2BP3 knockdown in K562 cells (chronic myeloid leukemia) does not induce apoptosis by itself, an observation we can also confirm for IGF2BP1 in tumor cells derived from gastrointestinal cancers (unpublished). However, IGF2BP3 knockdown enhances γ-irradiation-induced apoptosis by around 30 % in K562 cells [72]. This enhancement of apoptosis was largely abolished by supplementing recombinant IGF2, suggesting that IGF2BP3 may exert its protective effects essentially by promoting the expression of IGF2. In melanoma cells, knockdown of IGF2BP1 was also shown to be protective against chemotherapy-induced apoptosis [83]. Unfortunately, the role of p53 and involvement of the mitochondria in the observed apoptosis signaling was not investigated in the above studies, and remains an important area of enquiry. This is emphasized by reported observations in colon carcinoma-derived cells in which IGF2BP1 knockdown was proposed to induce apoptosis, as suggested on the basis of increased Caspase3/8 abundance as well as cleaved PARP and LaminA/C proteins [57].

### The role of IGF2BPs in cell migration

The identified target transcripts, in particular ACTB and CD44 (see Table 1), of IGF2BPs suggest a role of this protein family in controlling cytoskeletal organization, cell adhesion, and consequently cell migration. The most striking observation indicating a significant role of IGF2BPs in regulating cell motility was in *Xenopus* where the IGF2BP ortholog Vg1RBP/Vera promoted the directed migration of neuronal crest cells during development [27]. However, via which target mRNAs Vg1RBP/Vera modulates the migration of neural crest cells remains largely elusive .

The chicken ortholog of the human IGF2BP1, termed ZBP1 (Zipcode binding protein), was identified as a key regulator directing the localization of ACTB mRNA to the leading edge of fibroblasts as well as exploratory growth cones in primary neurons [2, 9, 84]. Although it remains unknown whether enhancement of neuronal crest cell migration by Vg1RBP/Vera also involves the localization of ACTB mRNA, these findings together indicated a pivotal role of IGF2BPs in modulating both cytoskeletal polarization and actin-driven cell migration. In support of this, IGF2BP1 was identified to control the spatially restricted translation of the ACTB mRNA in neuronal cells [6]. This suggested that the protein is an essential regulator of local ACTB monomer concentrations and thus F-actin polymerization, the driving force of cell protrusion. In developing mammalian neurons, the spatial control of ACTB protein levels by IGF2BPs or their orthologs is essentially involved in modulating neurite outgrowth and growth cone guidance [6, 43, 44]. Although actin remodeling and protrusion of growth cones is regulated by somewhat different mechanisms than observed in the migration of mesenchymal cells, IGF2BPs were also shown to enhance the migration of the latter. In tumor-derived cells, IGF2BPs were demonstrated to enhance the formation of lamellipodia, enforce intrinsic polarization, and thus promote directed cell migration [14, 61, 85, 86]. Although all these findings support the notion that IGF2BPs, in particular IGF2BP1, promote directed cell migration, it was unknown if this role was solely due to the spatiotemporal control of ACTB mRNA translation or involved the regulation of additional target mRNAs. However, recent studies by the Singer laboratory provide striking evidence that the localization of endogenous ACTB mRNA to the leading edge of fibroblasts lags behind the rapid change in migration directionality observed during random migration [87]. These findings suggest that the enhancement of ACTB mRNA localization sustains the directed migration in response to chemotactic cues rather than initiating cell protrusion. This obviously supports findings in neurons where IGF2BPs were suggested to support the guidance of growth cones during development [43, 44]. Despite this strong evidence indicating an essential role of IGF2BPs in the modulation of chemotactic movement, IGF2BP1 apparently also serves a role in controlling the random migration of tumor-derived cells. Our recent studies indicate that IGF2BP1 promotes the velocity of tumor cell migration and migration-supportive adhesion by limiting MAPK4 mRNA translation and consequently MK5-directed phosphorylation of HSP27 [14]. The latter is frequently upregulated in various cancers and is essentially involved in modulating cellular G-/F-actin ratios by an enhanced sequestering of ACTB monomers upon MK5-directed phosphorylation at two key serine residues [14]. Thus, by antagonizing MK5-directed phosphorylation of HSP27 and concomitantly limiting ACTB mRNA translation, IGF2BP1 serves as a ‘post-transcriptional fine tuner’ of ACTB monomer levels (reviewed in [88]). However, IGF2BP1 not only controls the speed of migration but also modulates intrinsic cell polarization, presumably via at least two target transcripts. The reported control of ACTB mRNA localization directs actin monomers to the site of active protrusion and thus determines a dynamic cytoskeletal polarization. Although this is presumably largely dispensable for randomly walking cells, it could have a severe impact on sustained motion during development or in chemotactic gradients [85, 87]. On the other hand, IGF2BP1 surprisingly enhances the expression of the tumor-suppressor PTEN and thereby shifts the cellular PIP3/PIP2 equilibrium [14]. This enhancement of PTEN expression enforces intrinsic cell polarization in a RAC1-dependent manner in vitro. Hence, in tumor-derived cells still expressing functional PTEN, IGF2BP1 can enhance both the speed and the directedness of cell movement. In glioblastoma-derived tumor-cells lacking PTEN, IGF2BP1 was found to exclusively promote the speed but not the directedness of random migration [14]. Despite conclusive evidence supporting IGF2BPs as key regulators of cell migration, their potential role in tumor cell invasion and metastasis remains poorly understood. However, it should be noted that the de novo synthesis of IGF2BP3 and to a lesser extent IGF2BP1 have been reported to correlate with enhanced metastasis and poor prognosis in various cancers. Moreover, the de novo synthesis of transgenic IGF2BP1 in mammary tissues of lactating mice induced both the formation of primary lesions as well as metastasis [62]. Consistently, IGF2BP1 and IGF2BP3 were shown to enhance the in vitro formation of invadopodia by promoting the expression of CD44 [15]. In agreement with this, we have observed that the forced expression of IGF2BP1 promoted the invasiveness of tumor cells in vitro, whereas the opposite was observed upon its knockdown (unpublished). Moreover, significant expression of IGF2BPs was observed in metastasizing colorectal carcinomas (CRC) with high expression of IGF2BPs at the invasive front [61]. Notably, IGF2BP expression apparently prevails during metastasis, since high levels of IGF2BPs were also observed in CRC-derived lymph node metastasis [61]. Although these studies fail to reveal which paralogues of the IGF2BP protein family potentially modulate the invasiveness of CRC, they support the view that IGF2BPs enhance the metastatic potential of tumor cells. In contrast, in vitro studies suggest that IGF2BP1 could interfere with metastasis by enhancing intrinsic cell polarization to a level which abolishes chemotactic responsiveness [85]. Surprisingly, IGF2BP1 depletion in mammary carcinoma-derived T47D cells was reported to enhance cell migration whereas the opposite was observed upon the overexpression of ZBP1, the chicken ortholog of IGF2BP1 [50]. These findings are puzzling, since we observe that IGF2BP1 promoted the migration of tumor-derived cells in vitro and enhanced cell polarization in a PTEN-dependent manner [14]. These observations are consistent with reports indicating IGF2BP1 to enhance cell polarization, as well as studies demonstrating that IGF2BPs promote cell migration and the formation of lamellipodia [61, 85, 86]. One simple explanation is that what is described to be IGF2BP1 in T47D is a specific IGF2BP1 mutant/isoform or another IGF2BP paralogue, since IGF2BP1 expression is barely observed in a panel of breast cancer-derived cells including T47D [48]. However, this does not explain why the overexpression of ZBP1 slows down T47D cell migration.

Despite controversial observations regarding a potential involvement of IGF2BPs in metastasis, IGF2BP1 and IGF2BP3 emerge as potent modulators of cell migration during development and in cancer. This role is likely to involve the spatiotemporal fine tuning of actin dynamics, the driving force of cell motility. Moreover, there is substantial evidence suggesting IGF2BPs modulate cell adhesion, the formation of invadopodia, and intrinsic cell polarization. Notably, IGF2BP2 could add to IGF2BP-directed control of cell migration, presumably by modulating cell adhesion. Recent reports suggest that IGF2BP2 controls the expression of proteins modulating cell matrix contact formation, LIMS2 and TRIM54, as well as the extracellular matrix protein LAMB2 [89, 90]. Hence, substantial in vitro and in particular in vivo studies are required to decipher how IGF2BPs modulate cell adhesion, migration, and most importantly metastasis. However, in view of the somewhat controversial observations reported, it appears likely that their role in metastasis is essentially determined by the cancer or cell type analyzed.

On a slightly different note, a recent publication has uncovered an unexpected role for IGF2BP1 in a mouse model of colon wound healing. IGF2BP1 was found to promote the expression of prostaglandin-endoperoxide synthase 2 (Ptgs2), presumably by preventing Ptgs2 mRNA degradation in colonic mesenchymal stem cells [91]. This was suggested to enable or enhance efficient wound closure, supporting a pivotal role of IGF2BPs in cell migration. Moreover, there is a hypothesis within the oncology field that has speculated that cancers are ‘wounds that never heal’ [92]. Recent papers on the subject have reported the importance of PTEN [93], IGFs, and MYC in these processes, significantly transcripts also regulated by IGF2BPs, and also that the majority of effected transcripts are shared in both wound healing and cancer. Further research is certainly required to elucidate further if IGF2BPs serve roles in the process of wound healing, and whether this role is exploited in tumors for growth and metastasis and may lead to the mechanisms of IGF2BP1/3 re-expression in adult tissues.

## Current limitations and concluding remarks

Descriptive studies of IGF2BPs demonstrate well-correlated expression throughout development and in reproductive tissues (which have high proliferation requirements). To date, there are few mechanistic comparative studies involving paralogues and isoforms within the IGF2BP family. This poses a significant limitation in deciphering the role of individual IGF2BPs in cancer. In contrast to IGF2BP1 and IGF2BP3, for which de novo synthesis in various malignancies has been reported, IGF2BP2 has been implicated as a candidate gene involved in type 2 diabetes (T2D) (reviewed in [30]). However, it has to be noted that, except for a role in IGF2 mRNA translation proposed to be regulated by mTORC1-directed phosphorylation of IGF2BP2, there is currently no functional evidence for a role of this paralogue in glucose homeostasis, insulin signaling, or diabetes [19]. The only evidence for a putative role of IGF2BP2 in T2D is provided by various studies correlating SNPs in the second intron of the IGF2BP2 locus with T2D. Notably, some studies correlated IGF2BP2-SNPs with reduced pancreatic β-cell function rather than with reduced insulin sensitivity (reviewed in [30]). This could indicate a role of IGF2BP2 and potentially its paralogues in pancreatic development and/or function. Supporting this assumption, loss-of-function studies in *Xenopus* revealed that Vg1RBP/Vera is involved in determining pancreatic cell fate during development [94]. Notably, we observed that IGF2BP2 and potentially the expression of IGF2BP3 were upregulated in old male mice (Fig. 3a). Hence, current evidence favors a role of IGF2BP2 in metabolic control and not in malignancy. This could point to a lack of research, rather than a lack of function, as there is little evidence towards it *not* being involved in malignancy either. It could be speculated that the family members act in balance to drive embryonic growth, with IGF2BP2 functioning as a cell survival and maintenance factor, unable to drive growth on its own, but nonetheless integral to aid growth in non-limited nutrient supply conditions in the embryo. This remains to be proven, but demonstrates the need for family members to be studied (where possible) within the same contexts. Multiple knockout/knock-in conditional mice studies are essential to determine which of the family members are required for carcinogenesis. It is relevant to note here that IGF2BP1 and IGF2BP3 were found to occupy the same mRNPs in one context, a finding supported by the observation that IGF2BPs could form homo- as well as hetero-dimers upon RNA-binding [3, 24, 95]. Although these findings provide strong evidence for cooperative regulation of mRNA fate by distinct paralogues, many cancer studies suggest that IGF2BPs could also act in an independent manner. As already eluded to, crossing of multiple IGF2BP paralogue knockouts would be advantageous in understanding the interactions and signaling effects, but first, formal characterization of conditional and tissue specific knock-out mice are required for each paralogue. The current models need to be improved. Transgenic mice (especially IGF2BP1 and 3) that replicate the re-expression observed in cancer pathology would be extremely useful for mechanistic studies, but also for anti-IGF2BP drug development and testing, in vivo. Notably, the only study addressing this aspect in mammary carcinomas revealed interesting differences of IGF2BP1 functions in vitro versus in vivo. For instance, IGF2BP1 expression in the mammary tissue of female mice led to an upregulation of IGF2 and H19 but not MYC mRNA levels [62].

Somewhat concerning is the specificity of currently available antibodies. Evidence within our laboratory has shown that development of paralogue specific antibodies is difficult (Fig. 3b; Fig. S4). Although we have achieved a significant paralogue specificity which allows for a largely unbiased analysis of IGF2BP expression in most cancer-derived cells, we currently cannot exclude slight paralogue cross-reactivity of monoclonal antibodies at high protein concentrations. Notably, we had no success with polyclonal peptide-directed antibodies, although other laboratories reported high paralogue specificity of their polyclonal antibodies [31, 58, 96]. This putative bias imposed by used antibodies is largely ignored, since many studies show specificity of used siRNA-mediated knockdown by western blotting, but, unfortunately, not all papers give evidence towards the specificity of their tools. The similarity in paralogue kDa size and amino acid sequence similarity makes differentiation of paralogues difficult by western blot, therefore confidence in antibodies and siRNAs is critical. With much of the research into IGF2BPs in the cancer context using immunohistochemistry, here, too, it is essential to generate and use paralogue-specific antibodies.

Even though IGF2BP1 and 3 have been demonstrated as putative targets for drug design for use as chemotherapy since the 1990s, there are no small molecules currently available for specific inhibition of IGF2BP function. Development of such compounds/molecules would have great therapeutic potential and also have a use towards mechanistic studies. Recent work on the IGF2BP1 protein structure has paved the way towards possible drug design, possibly through fragment-based screening or virtual ligand screening to inhibit binding of substrates such as the MYC or IGF2 mRNAs [4, 23]. However, structural analyses of all four KH-domains in complex with target RNAs are required for the development of specific compounds. The possibility of paralogue-specific transcript binding inhibition and/or specific-transcript binding inhibition is an exciting next stage for IGF2BP research.

Consistently, various studies indicate the IGF2BP family as powerful growth factors, critical in vertebrate development. Current evidence points to the more closely related IGF2BP1 and IGF2BP3 being pro-oncogenic and pro-migratory when re-expression is forced or induced in adult tissues, and to IGF2BP2 having a role in metabolic homeostasis and response to nutrients. More specific information is required as to the specific isoform and paralogue expression of significance in cancer etiology and patient outcome. Current literature highlights the close relationship between IGF2BP-dependent mechanisms in cell migration in both embryos and neoplasia. Future studies will hopefully bridge the gap in knowledge between in vitro mechanistic studies on cell migration and in vivo metastasis. Studies into IGF2BPs have shed light over the potential diversity and wide-reaching effects of individual RNA-binding proteins within cell homeostasis and cancer progression. More importantly, however, there is growing evidence indicating RNA-binding proteins, in particular IGF2BPs, as clinically significant markers and attractive targets for future anti-cancer/anti-metastatic drug design.

## Electronic supplementary material

Below is the link to the electronic supplementary material.

Supplemental Fig. 1. Enrichment of IGF2BP1 in cytoplasmic mRNP complexes. Sedimentation of IGF2BP1 was analyzed in HEK293 cells with or without stable transfection of Flag-ZBP1 by sucrose gradient centrifugation. Fractionation of indicated proteins was monitored by Western blotting. RNA sedimentation was monitored by a continuous UV_254_-spectrum. RPS6 (ribosomal protein S6) served as a control indicating the 48S, 80S and polysomal fractions. VCL (vinculin) served as a control for non-RNA associated proteins. Note, the exogenous Flag-ZBP1 is enriched in non-polysomal ‘lighter’ fractions suggesting association with small RNPs or non-RNA associated protein. In contrast, endogenous IGF2BP1 is enriched in 48-80S fractions with small amounts of the protein observed in polysomal fractions, as previously reported [31]. These observations indicate that RNA-binding studies using exogenous protein as a bait are likely biased due to aberrant sedimentation, presumably indicating altered protein-RNA association [compare to: [10, 22]].*Method:* HEK293 cells were stably transfected with Flag-ZBP1 using zeocin selection. Sucrose gradient centrifugation was essentially performed as previously described [11] (JPEG 156 kb)

Supplemental Fig. 2. All four KH-domains of IGF2BP1 modulate binding to RNA in vitro. **(A, B)** RNA-binding of recombinant ZBP1 and indicated mutant proteins was monitored by filter binding studies using Atto680 labeled in vitro transcribed RNAs. In mutant proteins, the GXXG-motif in indicated KH-domains was converted to GEEG to abolish RNA-binding. Upper panels: scheme of the ACTB and MYC mRNAs (Acc. No.) with RNA probes indicated in red. Numbers indicate nucleotide (nt) positions according to reference sequences indicated by accession numbers. Error bars indicate s.d. of three independent analyses. **(C)** Table indicating determined K_D_-values for binding of each protein to the ACTB or MYC bait respectively. Note, mutation of indicated di-domains affects RNA-binding in a substrate-dependent manner. Only mutation of all four KH-domains essentially abolishes binding. This indicates that all KH-domains of ZBP1, the chicken ortholog of human IGF2BP1, determine RNA-binding specificity and affinity in vitro. *Method:* ZBP1 mutant cDNAs were generated by site-directed mutagenesis and subcloned in pGEX6p1, essentially as previously described [6]. Protein purification, RNA in vitro transcription, filter binding and determination of K_D_-values was essentially performed as recently described [97] (JPEG 507 kb)

Supplemental Fig. 3. Alternative poly-adenylation sites in IGF2BP transcripts. Schematic of indicated IGF2BP transcripts (Acc. No.) with 5′-UTRs, open reading frame (ORF) and 3′-UTRs with alternative poly-adenylation sites depicted in red. Numbers indicate nucleotides (JPEG 147 kb)

Supplemental Fig. 4. IGF2BP expression in HEK293 cells. **(A)** HEK293 cells were transfected with IGF2BP1 (I1), IGF2BP2 (I2), IGF2BP3 (I3) or control siRNAs (C) for 72 h. IGF2BP expression was monitored by Western blotting of total cell lysates by indicated antibodies (right panel). IGF2BP paralogues are indicated (left panel) according to the nomenclature depicted in Fig. 1. Note, the IGF2BP3-directed monoclonal antibody shows a modest cross-reactivity with IGF2BP1 which was observed exclusively in HEK293 cells, since these express IGF2BP1 at severely upregulated levels compared to IGF2BP3. Three IGF2BP2 protein isoforms are observed in HEK293 cells. The monoclonal anti-IGF2BP2 antibody shows a strong cross-reactivity with an unknown protein (#) **(B)** IGF2BP2 isoforms were analyzed in U2OS cells stably transfected with control (C), pan-IGF2BP2 (I2-pan) or IGF2BP2-a (I2-a) specific shRNAs. Note, the IGF2BP-a directed shRNAs selectively depletes the longest and shortest protein isoforms. This fits well with the calculated molecular weights of the IGF2BP2 isoforms: IGF2BP2-a, ~ 66 kDa; IGF2BP2-b, ~ 61.8 kDa; IGF2BP-a*, ~ 58.6 kDa. IGF2BP2-a presents the predominant and longest IGF2BP2 isoform. The alternatively spliced isoform IGF2BP2-b as well as IGF2BP-a* are expressed at significantly lower levels. A fourth isoform resulting from leaky scanning of isoform IGF2BP2-b is not observed, presumably due to low expression of IGF2BP2-b transcript and a low frequency of leaky scanning observed for IGF2BP2-a.*Method:* HEK293 cells were cultured, transfected and analyzed essentially as previously described [6]. Mouse monoclonal antibodies were raised against recombinant IGF2BP full length proteins, as described in [11, 14]. SiRNA sequences: I1, UGAAUGGCCACCAGUUGGA; I2-pan, GGGAAGAUGUUAAGAUAUG; I3, UAAGGAAGCUCAAGAUAUA. Sh-RNA: I2-a, ACCAACAAGCCAAUCUGAUCC (JPEG 428 kb)

## Abbreviations

Acc. no.: Accession number
CRD: Coding region stability determinant
CRD-BP: Crd binding protein (IGF2BP gene alias)
dIMP: *Drosophila* IGF2BP
IGF2BP: Insulin-like growth factor 2 mRNA-binding protein
IMP: IGF2 mRNA binding protein (gene alias)
KH: hnRNP-K homology domain
PAR-CLIP: Photoactivatable ribonucleoside-enhanced crosslinking and immuno-precipitation
RBP: RNA-binding protein
RIP: RNA immunoprecipitation
RNP: Ribonucleoprotein (granule)
RRM: RNA-recognition motif
T2D: Type 2 diabetes
VICKZ: *V*g1RBP/Vera *I*GF2BP *C*RD-BP *K*OC *Z*BP1 (gene family alias)
