# Supplementary figures and images for: Insulin-like growth factor 2 mRNA-binding proteins (IGF2BPs): post-transcriptional drivers of cancer progression?
Source: Cell Mol Life Sci. 2012 Oct 16;70(15):2657–75. doi: 10.1007/s00018-012-1186-z (PMC3708292; doi:10.1007/s00018-012-1186-z)

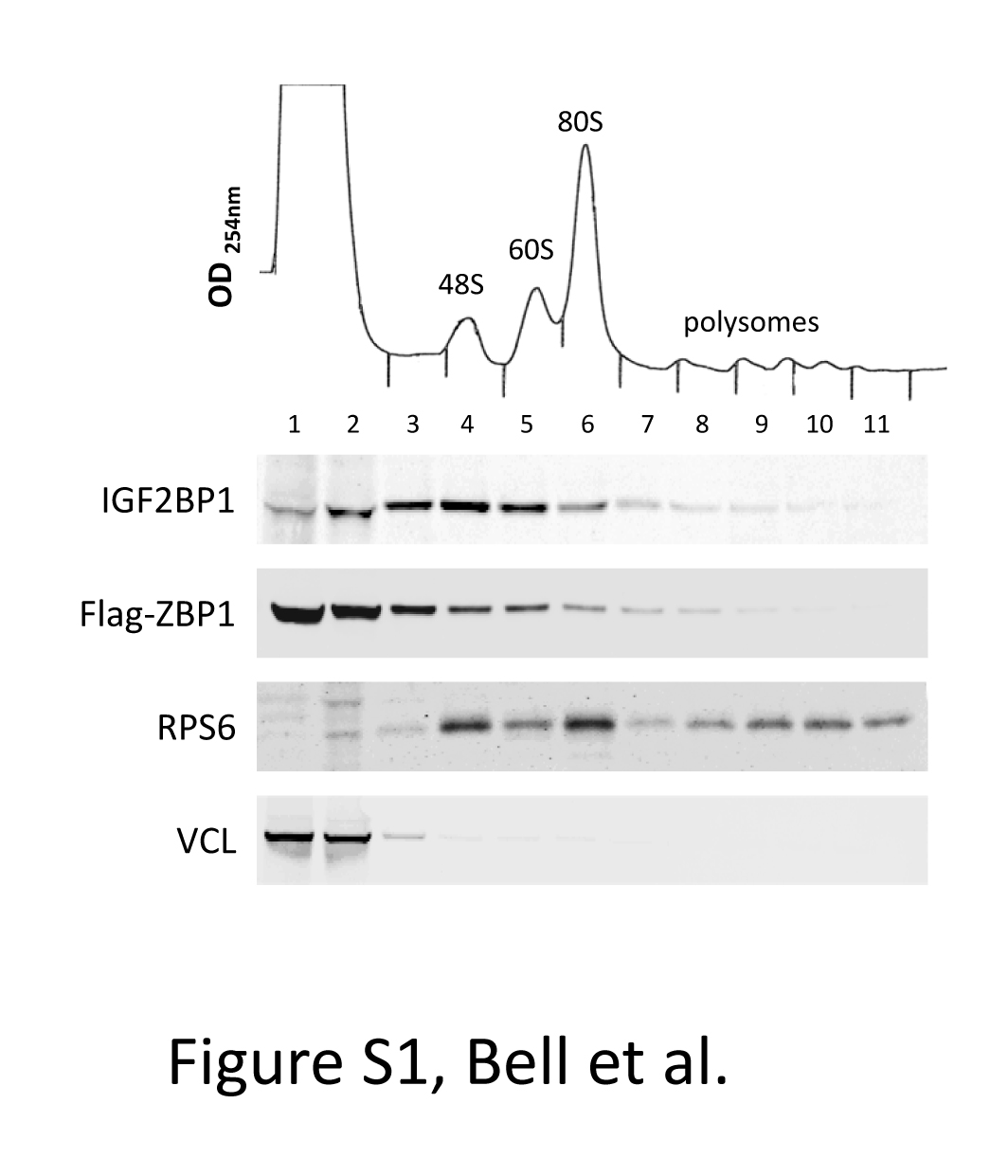

Supplement: Supplementary file 1 — Supplemental Fig. 1. Enrichment of IGF2BP1 in cytoplasmic mRNP complexes. Sedimentation of IGF2BP1 was analyzed in HEK293 cells with or without stable transfection of Flag-ZBP1 by sucrose gradient centrifugation. Fractionation of indicated proteins was monitored by Western blotting. RNA sedimentation was monitored by a continuous UV254-spectrum. RPS6 (ribosomal protein S6) served as a control indicating the 48S, 80S and polysomal fractions. VCL (vinculin) served as a control for non-RNA associated proteins. Note, the exogenous Flag-ZBP1 is enriched in non-polysomal ‘lighter’ fractions suggesting association with small RNPs or non-RNA associated protein. In contrast, endogenous IGF2BP1 is enriched in 48-80S fractions with small amounts of the protein observed in polysomal fractions, as previously reported [31]. These observations indicate that RNA-binding studies using exogenous protein as a bait are likely biased due to aberrant sedimentation, presumably indicating altered protein-RNA association [compare to: [10, 22]].Method: HEK293 cells were stably transfected with Flag-ZBP1 using zeocin selection. Sucrose gradient centrifugation was essentially performed as previously described [11] (JPEG 156 kb) [file 18_2012_1186_MOESM1_ESM.jpg]

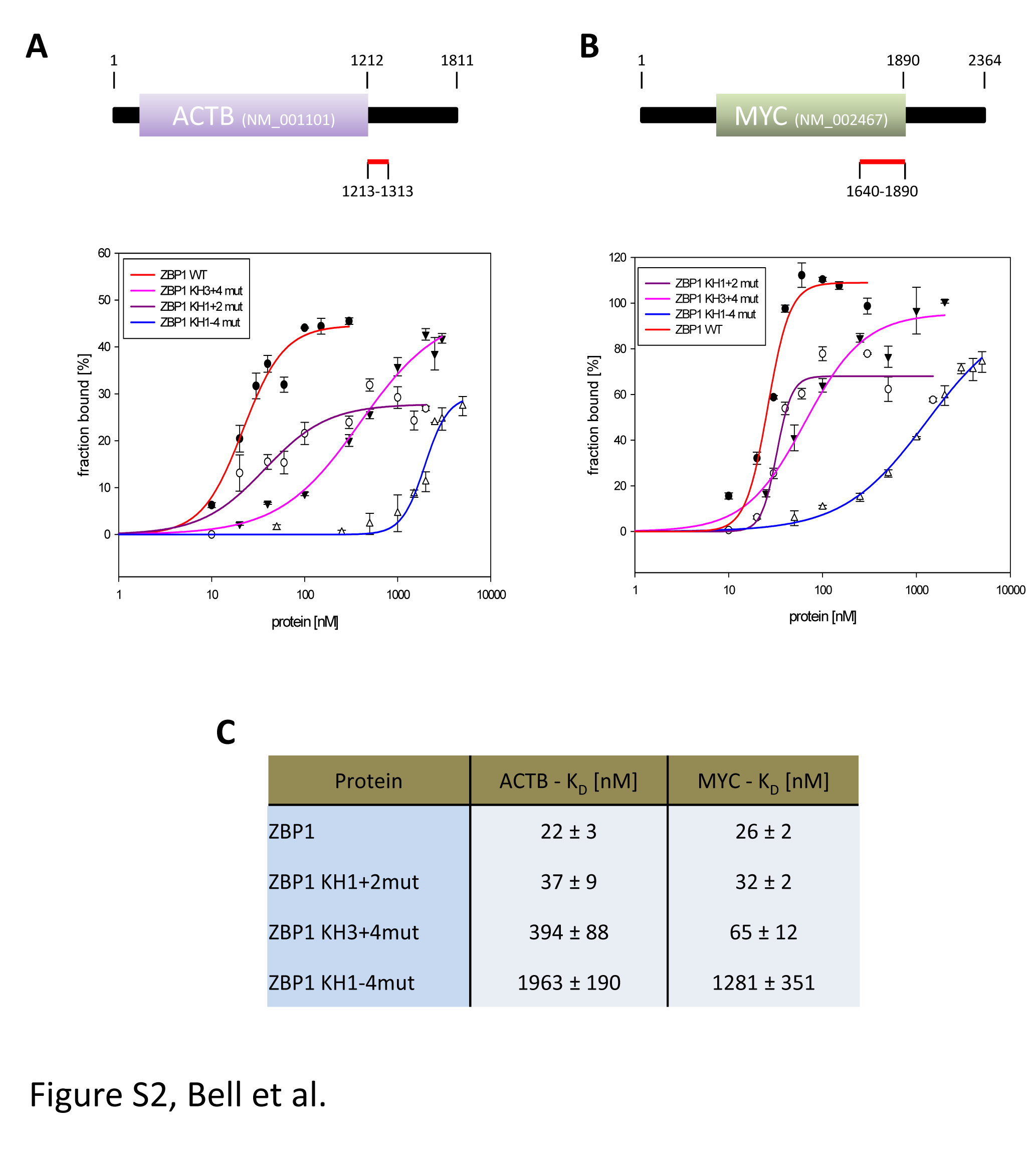

Supplement: Supplementary file 2 — Supplemental Fig. 2. All four KH-domains of IGF2BP1 modulate binding to RNA in vitro. (A, B) RNA-binding of recombinant ZBP1 and indicated mutant proteins was monitored by filter binding studies using Atto680 labeled in vitro transcribed RNAs. In mutant proteins, the GXXG-motif in indicated KH-domains was converted to GEEG to abolish RNA-binding. Upper panels: scheme of the ACTB and MYC mRNAs (Acc. No.) with RNA probes indicated in red. Numbers indicate nucleotide (nt) positions according to reference sequences indicated by accession numbers. Error bars indicate s.d. of three independent analyses. (C) Table indicating determined KD-values for binding of each protein to the ACTB or MYC bait respectively. Note, mutation of indicated di-domains affects RNA-binding in a substrate-dependent manner. Only mutation of all four KH-domains essentially abolishes binding. This indicates that all KH-domains of ZBP1, the chicken ortholog of human IGF2BP1, determine RNA-binding specificity and affinity in vitro. Method: ZBP1 mutant cDNAs were generated by site-directed mutagenesis and subcloned in pGEX6p1, essentially as previously described [6]. Protein purification, RNA in vitro transcription, filter binding and determination of KD-values was essentially performed as recently described [97] (JPEG 507 kb) [file 18_2012_1186_MOESM2_ESM.jpg]

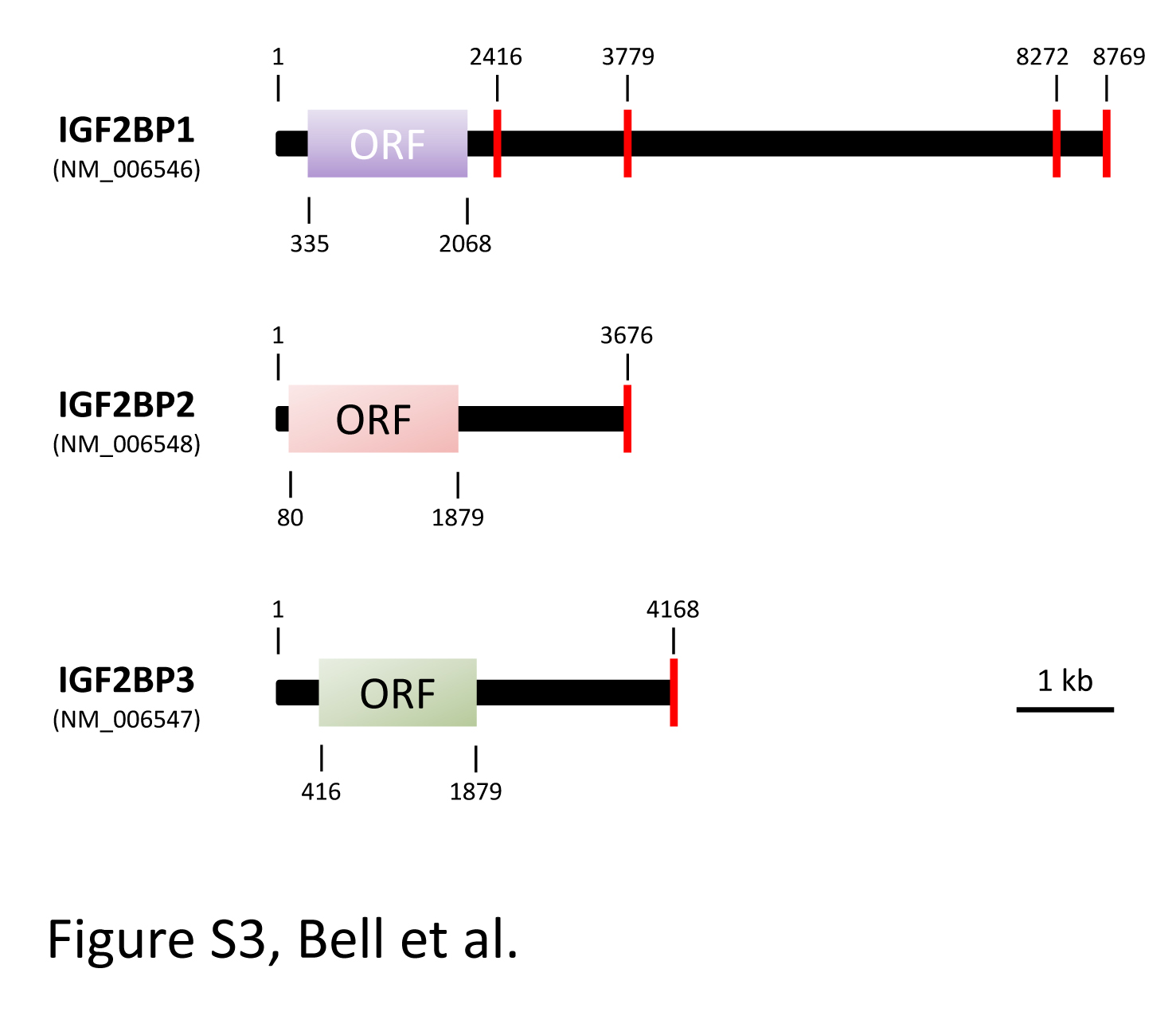

Supplement: Supplementary file 3 — Supplemental Fig. 3. Alternative poly-adenylation sites in IGF2BP transcripts. Schematic of indicated IGF2BP transcripts (Acc. No.) with 5′-UTRs, open reading frame (ORF) and 3′-UTRs with alternative poly-adenylation sites depicted in red. Numbers indicate nucleotides (JPEG 147 kb) [file 18_2012_1186_MOESM3_ESM.jpg]

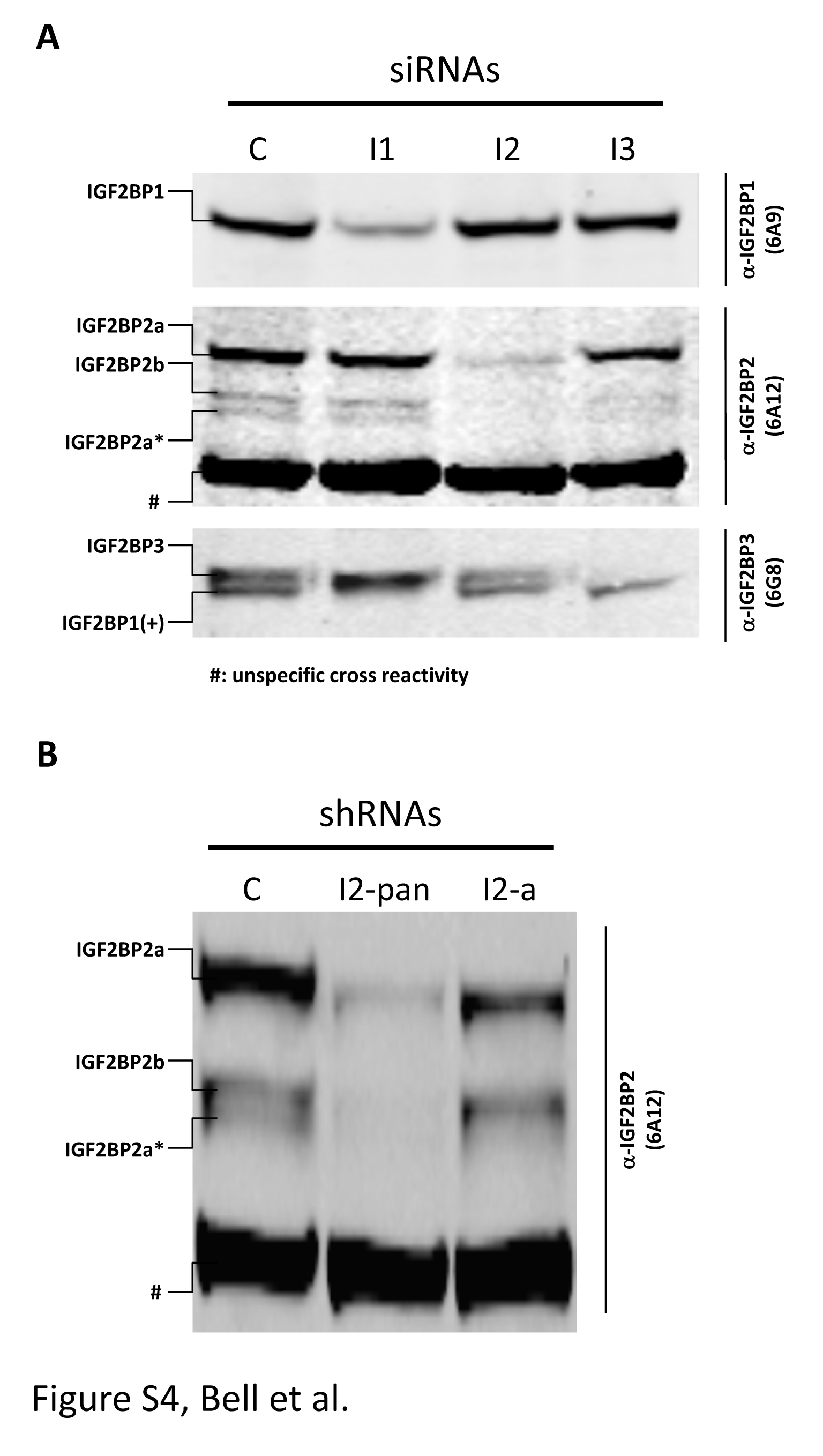

Supplement: Supplementary file 4 — Supplemental Fig. 4. IGF2BP expression in HEK293 cells. (A) HEK293 cells were transfected with IGF2BP1 (I1), IGF2BP2 (I2), IGF2BP3 (I3) or control siRNAs (C) for 72 h. IGF2BP expression was monitored by Western blotting of total cell lysates by indicated antibodies (right panel). IGF2BP paralogues are indicated (left panel) according to the nomenclature depicted in Fig. 1. Note, the IGF2BP3-directed monoclonal antibody shows a modest cross-reactivity with IGF2BP1 which was observed exclusively in HEK293 cells, since these express IGF2BP1 at severely upregulated levels compared to IGF2BP3. Three IGF2BP2 protein isoforms are observed in HEK293 cells. The monoclonal anti-IGF2BP2 antibody shows a strong cross-reactivity with an unknown protein (#) (B) IGF2BP2 isoforms were analyzed in U2OS cells stably transfected with control (C), pan-IGF2BP2 (I2-pan) or IGF2BP2-a (I2-a) specific shRNAs. Note, the IGF2BP-a directed shRNAs selectively depletes the longest and shortest protein isoforms. This fits well with the calculated molecular weights of the IGF2BP2 isoforms: IGF2BP2-a, ~ 66 kDa; IGF2BP2-b, ~ 61.8 kDa; IGF2BP-a*, ~ 58.6 kDa. IGF2BP2-a presents the predominant and longest IGF2BP2 isoform. The alternatively spliced isoform IGF2BP2-b as well as IGF2BP-a* are expressed at significantly lower levels. A fourth isoform resulting from leaky scanning of isoform IGF2BP2-b is not observed, presumably due to low expression of IGF2BP2-b transcript and a low frequency of leaky scanning observed for IGF2BP2-a.Method: HEK293 cells were cultured, transfected and analyzed essentially as previously described [6]. Mouse monoclonal antibodies were raised against recombinant IGF2BP full length proteins, as described in [11, 14]. SiRNA sequences: I1, UGAAUGGCCACCAGUUGGA; I2-pan, GGGAAGAUGUUAAGAUAUG; I3, UAAGGAAGCUCAAGAUAUA. Sh-RNA: I2-a, ACCAACAAGCCAAUCUGAUCC (JPEG 428 kb) [file 18_2012_1186_MOESM4_ESM.jpg]
